# Escape from humoral immunity is associated with treatment failure in HIV-1-infected patients receiving long-term antiretroviral therapy

**DOI:** 10.1038/s41598-017-05594-5

**Published:** 2017-07-24

**Authors:** Yabo Ouyang, Qianqian Yin, Wei Li, Zhenpeng Li, Desheng Kong, Yanling Wu, Kunxue Hong, Hui Xing, Yiming Shao, Shibo Jiang, Tianlei Ying, Liying Ma

**Affiliations:** 1 State Key Laboratory of Infectious Disease Prevention and Control, National Center for AIDS/STD Control and Prevention (NCAIDS), Chinese Center for Disease Control and Prevention, Beijing, China and Collaborative Innovation Center for Diagnosis and Treatment of Infectious Diseases, Hangzhou, China; 2 Key Laboratory of Medical Molecular Virology of MOE/MOH, School of Basic Medical Sciences, Fudan University, Shanghai, China; 3 Protein Interactions Section, Cancer and Inflammation Program, Center for Cancer Research, National Cancer Institute, National Institutes of Health, Frederick, Maryland USA; 4 Lindsley F. Kimball Research Institute, New York Blood Center, New York, USA; 5 Beijing You’an Hospital, Capital Medical University, Beijing, China and Beijing Institute of Hepatology, Beijing, China

## Abstract

Interindividual heterogeneity in the disease progression of HIV-1-infected patients receiving long-term antiretroviral therapy suggests that some host-related factors may have limited treatment efficacy. To understand the nature of factors contributing to treatment failure, we performed a retrospective cohort study of 45 chronically HIV-1-infected individuals sharing a similar demographics and route of infection, compared the differences between virologically suppressed (VS) and treatment failure (TF) patients with respect to clinical, immunological and virological characteristics. We found that the baseline diversity of HIV-1 env quasispecies was the major difference between VS and TF group, and higher baseline diversity in TF patients. We further predicted TF-related env mutations using a selection pressure-based approach, followed by an analysis of these mutations based on the available three-dimensional structures of gp120/gp41 or their complexes with neutralizing antibodies. Notably, almost all of the identified residues could be mapped to the epitopes of known HIV-1 neutralizing antibodies, especially the epitopes of broadly neutralizing antibodies, and these mutations tended to compromise antibody-antigen interactions. These results indicate that the escape of HIV-1 from host humoral immunity may play a direct role in TF in long-term antiretroviral-experienced patients and that based on env gene sequence of the viruses in the patients.

## Introduction

Rapid replication dynamics and high mutation rate of human immunodeficiency virus type 1 (HIV-1) allows the virus to evolve continuously and quickly during the course of infection^1^. With the advent of highly active antiretroviral therapy (ART), a combination of three or more potent antiretroviral drugs, profound suppression of viral replication has been achieved, and HIV-1-associated mortality has been dramatically reduced^2, 3^. Nonetheless, considerable variation of the virus is observed among patients in response to treatment, with a substantial proportion of individuals experiencing treatment failure, including virologic failure, i.e., inability to achieve or maintain suppression of viral replication; immunologic failure, i.e., failure of CD4 count to increase; and subsequent clinical failure^4, 5^. This interindividual heterogeneity in disease progression suggests that some host-related factors may have affected the effectiveness of ART. An understanding of such contributing factors associated with treatment failure is critical to developing an effective long-term HIV treatment strategy.

Host immune response elicited by HIV-1 contributes to the repression of viral replication, but it varies greatly in different individuals^6^. Some HIV-1-infected individuals are able to develop heavily mutated, broadly neutralizing antibodies (bnAbs) capable of neutralizing a broad spectrum of HIV-1 isolates^7–10^. These bnAbs only appear after several years of HIV-1 infection, suggesting the continuous viral escape and selection by autologous neutralizing antibodies^11, 12^. It is also intriguing to observe that a small group of HIV-1-infected individuals (<1%), termed elite controllers, spontaneously maintain undetectable levels of viral replication in the absence of antiretroviral therapy by mounting robust immune responses directed at HIV-1^13, 14^. Moreover, the escape mutants in the envelope glycoprotein (Env) during early HIV-1 infection primarily involved changes in N-linked glycosylation that could sterically inhibit the accessibility of neutralizing epitopes^11, 15^. All these findings suggest a critical role of selective pressure exerted by host humoral immune response in driving HIV-1 evolution. However, in ART-treated patients, the selective pressure is predominantly governed by antiretroviral drugs, and the effects of HIV-1 evolution and escape from host immune selective pressure on disease progression under long-term ART are largely unknown.

Therefore, to identify host and viral factors associated with differing clinical outcomes in HIV-1-infected patients receiving long-term antiretroviral treatment, we performed a retrospective cohort study of 45 chronically HIV-1-infected individuals sharing a similar demographic and ethnic background. All these patients were infected with HIV-1 subtype B’ through a similar route of infection, and they were enrolled in the National Free Antiretroviral Treatment Program in Anhui and Henan provinces, China. These patients were categorized into two groups: virologically suppressed (VS, n = 25) and treatment failure (TF, n = 20), according to disease outcome. The differences between the two groups with respect to clinical, immunological and virological characteristics were then compared. Interestingly, diversity of HIV-1 *env* quasispecies was found to be the major difference between the two groups. More specifically, compared to those in individuals in the VS group, both gp120 and gp41 gene quasispecies of HIV-1 in the TF patients showed higher baseline diversity. Treatment failure-related adaptive mutations were further predicted by using a selection pressure-based analysis of single-genome amplification (SGA)-derived full-length HIV-1 *env*. Notably, most of the identified mutations could be mapped to epitopes of known HIV-1 neutralizing antibodies, especially epitopes of bnAbs. These results demonstrate that the escape of HIV-1 from host humoral immunity contributes to disease progression in patients on long-term ART.

## Results

### Population characteristics

All 45 enrolled patients, who participated in a multicenter AIDS Cohort Study in Anhui and Henan provinces in China from 2004 to 2011, shared a common ethnic background and a similar route of infection, namely plasma donation. Similarity of immunological and viral genetic backgrounds provided a unique opportunity to explore the relationship between viral evolution and disease progression in different individuals.

Patients were divided into two groups according to disease outcome. The virologic suppression (VS) group was defined as viral load persistently <400 copies/ml after ART for 6 months. The treatment failure (TF) group was defined as repeated viral load >400 copies/ml after 6 months. By the close of the study period, 20 patients were classified as TF, and 25 patients were classified as VS. Baseline demographic and clinical features of all patients are shown in Table 1. The age, gender, high-risk behavior, and initial regimen showed no significant difference between VS and TF groups. Neither viral load nor CD4^+^ cell count differed between the two groups before treatment. Similar to the TF group, drug-resistant mutations in the pol region of the VS group were rarely found (P = 0.309, data not shown). Although there is a significant difference in switching regimens among the treatment failure cases, it occurred after the “treatment failure”.

**Table 1:** Demographic and clinical characteristics of HIV-1 subtype B′- infected patients.

	VS (n = 25)	TF (n = 20)	P Value	All Patients (n = 45)
Age, median years (Range)^a^	37 (26–64)	40 (30–56)	0.272	38 (26–64)
Male, No. (%)^b^	7 (28.00)	9 (45.00)	0.236	16 (35.56)
High-risk behavior, No. (%)^c^			1.000	
Former plasma donation	24 (96.00)	20 (100.00)		44 (97.78)
Sex	1 (4.00)	0 (0.00)		1 (2.22)
Initial Regimen, No. (%)^c^			0.314	
AZT + NVP + 3TC	13 (52.00)	12 (60.00)		25 (55.56)
D4T + NVP + 3TC	12 (48.00)	6 (30.00)		18 (40.00)
D4T + EFV + 3TC	0 (0.00)	1 (5.00)		1 (2.22)
AZT + EFV + 3TC	0 (0.00)	1 (5.00)		1 (2.22)
Viral Load at baseline, median log_10_copies/ml (Range)^a^	4.84 (3.21–6.98)	5.20 (4.07–5.98)	0.568	5.07 (3.21–6.98)
CD4^+^ count at baseline, median cells/μl (Range)^a^	166 (15–515)	247 (83–459)	0.110	219 (15–515)
Treatment time, median months (Range)^a^	60 (16–72)	63 (26–72)	0.670	63 (16–72)
Have switched regimens, No. (%)^c^	0 (0.00)	5 (25.00)	0.013	5 (11.11)
Have missed ART dose in last 7 days, No. (%)^c^	0 (0.00)	3 (15.00)	0.080	3 (6.67)
Have related drug-resistant mutation at baseline, No. (%)^c^	1 (4.00)	3 (15.00)	0.309	4 (8.89)

^a^Mann-Whitney Test; ^b^Chi-Square Test; ^c^Fisher’s Exact Test; Abbreviations: 3TC, lamivudine; AZT, zidovudine; d4T, stavudine; EFV, efavirenz; NVP, nevirapin.

**Figure 1:**
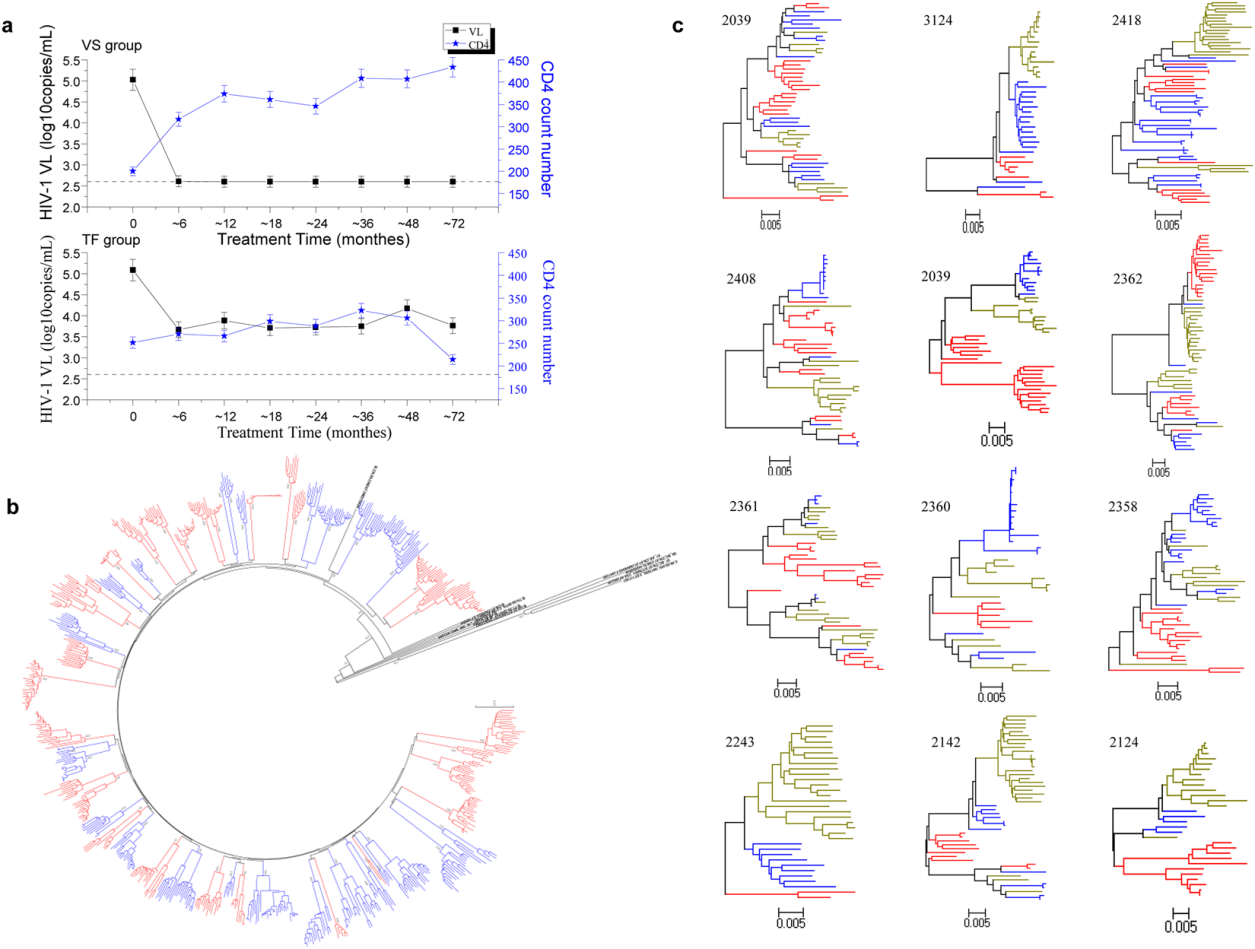
(**a**) Longitudinal changes in viral load (square) and HIV-1 CD4^+^ T cell count (pentacle) for VS (upper panel) and treatment failure TF (upper panel) during ART. HIV-1 vial load level of all VS was undetectable during ART, while the viral load levels of most TF fluctuated widely during ART, albeit lower than baseline viral load. Data are presented in mean ± standard deviation (SD) from three replicates. (**b**) Phylogenetic trees of 638 gp160 sequences at baseline between VS and TF groups. Each color represents one group (red: VS; blue: TF). Bootstrap values >85% are shown. No sequences showed evidence of cross-contamination between study subjects or contamination with laboratory strains. As expected, all sequences for each patient were clustered together. (**c**) Phylogenetic analysis of 552 gp160 amplicons, including 169 sequences from baseline, derived from 12 TF patients (red: baseline; blue: when viral load reached a level of >400 copies/ml; yellow: the maximum value of viral load).

Most individuals displayed declining trends in viral load and increased CD4^+^ cells after 6 months of ART (Fig. 1a). In the VS group, viral load levels remained at an undetectable level (≤400 copies/ml) after ART for 6 months (median treatment duration of 60 months). However, viral load in the TF group fluctuated widely, and detectable viral load was found during ART (median treatment duration of 63 months).

### Env sequences and phylogenetic relationship

For an accurate representation of HIV-1 *env* quasispecies *in vivo*, we used nested PCR single-genome amplification (SGA) to amplify the full-length *env* genes as previously reported^16^, whereby PCR products were derived from a single template molecule. Plasma collected at baseline and during ART was used to evaluate *env* clonal genotypes. A total of 1,021 single-genome full-length gp160 amplicons were obtained for cross-sectional and longitudinal analysis by SGA. Among these, 638 sequences derived from plasma of VS (n = 352) and TF (n = 286) at baseline were used for cross-sectional analysis. Analysis of gp160 nucleotide sequences at baseline showed that all sequences clustered phylogenetically with subtype B’ reference sequences (Fig. 1b). A neighbor-joining phylogenetic tree, including all sequences, showed no evidence of cross-contamination between study subjects or contamination with laboratory strains. As expected, the sequences for each patient were clustered together and formed monophyletic groups, except subjects 2124 and 3594 who were infected with two unrelated HIV-1 variants of subtype B’.

A total of 552 single-genome full-length gp160 amplicons, including 169 sequences from baseline, derived from plasma of 12 TF patients, who had never switched regimens and had good adherence until the last follow-up point, were used for longitudinal analysis (Fig. 1c). TF patients were included to compare *env* diversity between baseline (T0) and during ART. Sequence diversity was calculated using the Kimura 2-parameter model option in MEGA 5. The number of sequences of 12 patients included in the analysis was shown in Table S3. During ART, gp160 nucleotide sequences were crossed with each other or formed a separate cluster according to treatment time.

### Genetic characteristics of HIV-1 *env*

**Figure 2:**
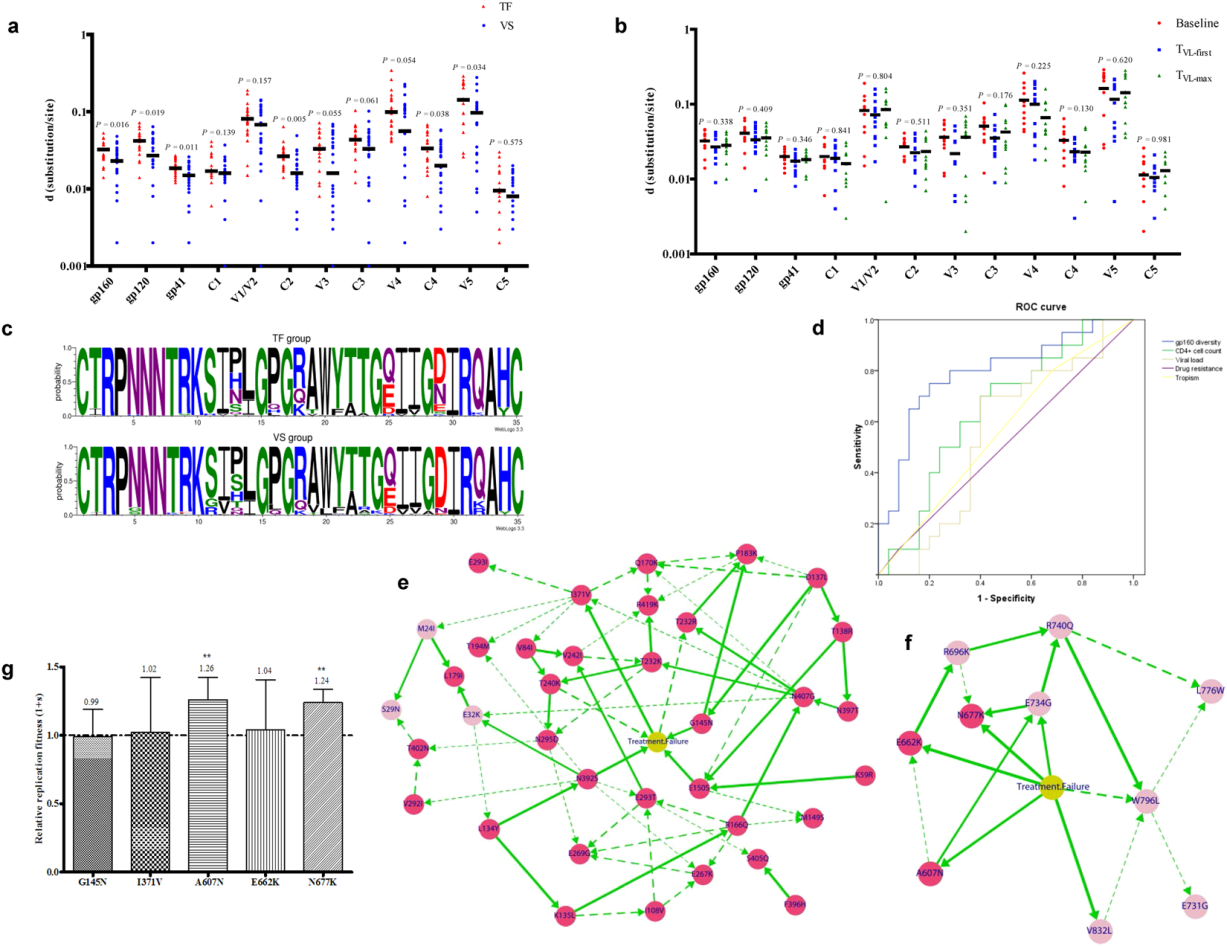
Comparison of *env* nucleotide diversity between patients with virologic suppression (VS) and treatment failure (TF) at baseline (**a**) and comparison of *env* nucleotide diversity from 12 patients with TF at three time points (**b**) (T_0_: baseline; T1: when viral load reached a level of >400 copies/ml; T_2_: the maximum value of viral load). The short lines represent the median value. P values were calculated by nonparametric Mann-Whitney test. (**c**) Consensus V3 sequences of VS group and TF group. The values on the y-axis indicate the percentage of each site, and relative height of each amino acid reflects its percentage at that site. (**d**) Logistic regression and receiver operating characteristic (ROC) analysis were performed to develop a predictive model and predictive value of HIV-1 *env* diversity for therapy outcome. Among the covariates including CD4+ T cell count, viral load, gp160 diversity, drug-resistant mutations and co-receptor usage, diversity of gp160 was the best single variable for predicting TF for 75.6% of individuals in this cohort, and an area under the curve (AUC) of 0.794 (CI95%, 0.657–0.931) was obtained in the ROC curve. Bayesian network analysis was used to screen therapy outcome-related putative adaptive mutations. Four mutation sites (G145N, E150S, I371V and N392S) of gp120 (**e**) and five mutation sites (A607N, E662K, N677K, E734G and V832L) of the gp41 region (**f**) were further predicted using Bayesian network. The width of edge was proportional to bootstrap support. The solid edges were considered to be robust and had bootstrap support over 65%, while dashed edges had bootstrap support between 35% and 65%. (**g**) Relative replication fitness of outcome-related putative adaptive mutants. Each mutant virus was competed against the wild-type in PM1 cells. A fitness value of 1.0 indicated that fitness was equal to that of wild type. Each bar represented the mean “1 + s value” ± SD from three replicates. Significant differences between mutant virus and wild-type virus were marked with asterisks (**p < 0.01).

Demographic characteristics and baseline drug-resistant mutations were the same between the VS and TF groups; therefore, we sought to investigate the difference between the two groups based on the diversity of HIV-1 *env* quasispecies. Quasispecies heterogeneity was quantitated by the mean genetic distance (d) of the sequences in each group. As shown in Fig. 2a, a relatively high level of heterogeneity occurred within V1-V2, V4 and V5 regions of gp120. The TF group exhibited higher baseline diversity in both gp120 (p = 0.019) and gp41 (p = 0.011) compared to that of the VS group. Interestingly, the most significant difference between the diversity of VS and TF groups occurred in the gp120 C2 region (p = 0.005).

In the longitudinal dataset, TF patients who never switched regimens were included to compare *env* diversity between baseline (T_0_) and during ART. Following baseline, two time points were selected based on viral load level: 1) when viral load reached a level >400 copies/ml (T_1_) and 2) maximum value of viral load (T_2_). A total of 536 gp160 sequences derived from 12 TF patients were analyzed and displayed no significant difference between baseline, T_1_ and T_2_ in gp160, or its subregion diversity (Fig. 2b). Although the TF group had a higher viral diversity at baseline, these results indicate that the viral *env* quasispecies did not further increase in these patients during ART.

We also analyzed the length of amino acid sequences and the number of potential N-linked glycosylation sites (PNGs) of HIV-1 *env* at baseline. No evident difference was seen in either length of amino acid sequence or number of PNGs in gp120 between the VS and TF groups (Table S1). Although the TF group exhibited relatively shorter length of amino acid sequence in gp41, the number of PNGs was no different from that of the VS group. Overall, no substantive difference was observed in gp160 between the two groups with respect to either length of amino acid sequence or number of PNGs.

The gp120 V3 loop has been suggested as a major viral determinant for co-receptor preference^17^. Thus, we also compared the V3 loops of the VS and TF groups. As shown in Fig. 2c, no evident difference was detected in the charge and amino acid usage of V3 loops between the two groups. The two groups also exhibited similar tetramer compositions of the tip of V3 loops, typically GPGR and GPGQ.

To evaluate variables that have contributed to treatment outcome at baseline, a logistic regression analysis was performed. Variables, including CD4^+^ T cell count, viral load, gp160 diversity, drug-resistant mutations and co-receptor usage, were all pooled for analysis. Interestingly, gp160 diversity was the only variable that remained significant in the final model, and it was the best single variable for predicting TF for 75.6% individuals in this cohort (Fig. 2d). An area under the curve (AUC) of 0.794 (95% confidence interval 0.657–0.931) was obtained in the ROC curve, with a sensitivity and positive predictive value of 75.0% at a 0.50 cutoff and a specificity and negative predictive value of 80.0%. Taken together, these results suggest that higher gp160 diversity significantly associated to the risk of treatment failure.

### Determination of treatment failure-related HIV-1 *env* adaptive mutation

To gain more insight into the difference in *env* quasispecies diversity between VS and TF groups, a selection pressure-based analysis was performed to investigate patterns of *env* evolution. The ratios of non-synonymous (Ka) and synonymous (Ks) nucleotide substitutions were calculated, as described in the Methods section, and used as an indicator of selective pressure acting on Env-coding genes. Treatment failure-related adaptive mutations were screened by the Ka/Ks ratio of individual residues as follows: (a) Ka/Ks > 1 and sequence frequency > 0.01 in the TF group, while Ka/Ks < 1, or Ka/Ks > 1, but LOD < 2, in the VS group; (b) sequence frequency of TF group higher than that of VS group (p < 0.05), while the corresponding population frequency of the TF group was over 0.15.

In total, 36 and 10 putative adaptive mutations related to treatment failure from gp120 and gp41 regions were obtained, respectively (Table 2). Among them, 4 critical mutations (G145N, E150S, I371V and N392S) of gp120 (Fig. 2e) and 5 critical mutations (A607N, E662K, N677K, E734G and V832L) of gp41 (Fig. 2f) were further predicted to be directly related to treatment failure using Bayesian network. The G145N, I371V, A607N, E662K and N677K mutations still existed with high frequency during ART (Table S2). No mutations were observed in the N-terminal heptad repeat (NHR) region (residues 542–592) of gp41, the target site of the HIV-1 fusion inhibitors, such as enfuvirtide (also known as T20) derived from the gp41 C-terminal heptad repeat (CHR) region^18^.

**Table 2:** Therapy failure-related adaptive mutations.

Mutation^a^	bnAbs	Population frequency		Region^a^	TF group		VS group	
		TF group	VS group		Ka/Ks	Sequence frequency	Ka/Ks	Sequence frequency
K59R	—	0.15	0.04	C1	24	0.08	0.75	0.04
V84I	—	0.2	0.04	C1	30.95	0.06	0.25	0
I108V	—	0.2	0.08	C1	17.27	0.08	2.18	0.01
L134Y	PGT122	0.2	0.04	V1-V2	7.2	0.13	0.82	0.03
K135L		0.15	0.04	V1-V2	7.67	0.08	0.35	0.03
D137L		0.15	0	V1-V2	27	0.09	—	—
T138R		0.15	0	V1-V2	21	0.07	—	—
G145N		0.5	0.08	V1-V2	58	0.2	0.67	0.01
M149S		0.15	0	V1-V2	4	0.01	—	—
E150S		0.15	0	V1-V2	40	0.14	—	—
R166Q	PG9, PG16	0.15	0	V1-V2	25	0.09	—	—
Q170K		0.15	0	V1-V2	72.26	0.09	—	—
L179I	830A	0.15	0	V1-V2	138.6	0.1	—	—
P183K		0.15	0	V1-V2	30	0.1	—	—
T194M		0.2	0.04	V1-V2	7	0.02	2	0.01
T232K	35O22	0.3	0.16	C2	3.87	0.18	0.97	0.08
T232R		0.15	0	C2	2.43	0.11	—	—
T240K		0.15	0.04	C2	71.69	0.1	2.38	0
V242I		0.15	0.04	C2	1.99	0.08	0.04	0
E267K		0.15	0.04	C2	1.7	0.06	0.5	0
E269G		0.25	0.12	C2	2.27	0.09	0.69	0.05
V292I	B12	0.25	0.08	C2	2.46	0.04	1.04	0.01
E293T		0.15	0.04	C2	23	0.08	0.5	0
E293I		0.2	0.04	C2	7	0.02	0.5	0
N295D		0.3	0.04	C2	3.5	0.07	0.33	0
I371V	VRC01	0.55	0.04	C3	27.65	0.21	1.36	0
N392S	PGT135	0.2	0.04	V4	1.18	0.14	0.03	0
F396H		0.15	0	V4	14	0.05	—	—
N397T		0.2	0	V4	63.93	0.08	—	—
T402N		0.2	0.04	V4	47.79	0.04	14.27	0.01
S405Q		0.15	0.04	V4	1.18	0.05	0.1	0.01
N407G		0.5	0.28	V4	1.57	0.25	0.98	0.14
R419K	B12	0.3	0.08	C4	1.14	0.13	0.55	0.03
A607N	—	0.15	0	gp41	46	0.16	—	—
E662K	2F5	0.35	0	gp41	61	0.21	—	—
N677K	4E10	0.8	0.44	gp41	3.33	0.61	0.94	0.27

^a^HXB2 Env was used as a reference sequence.—: no associated bnAbs.

Afterwards, using a flow cytometry-based multiple-cycle growth competition assay, as described previously^19^, we also measured the relative replication fitness of several treatment failure-related Env adaptive mutations, including G145N, I371V, A607N, E662K and N677K mutant viruses. Although the 1 + s fitness values of A607N and N677K increased slightly, the 1 + s fitness values of G145N, I371V and E662K viruses were not significantly different from those of the wild-type, suggesting that the change in viral fitness of these variants may not be a major determinant of treatment outcome (Fig. 2g).

### Mapping of treatment failure-related mutations on HIV-1 Env structure

**Figure 3:**
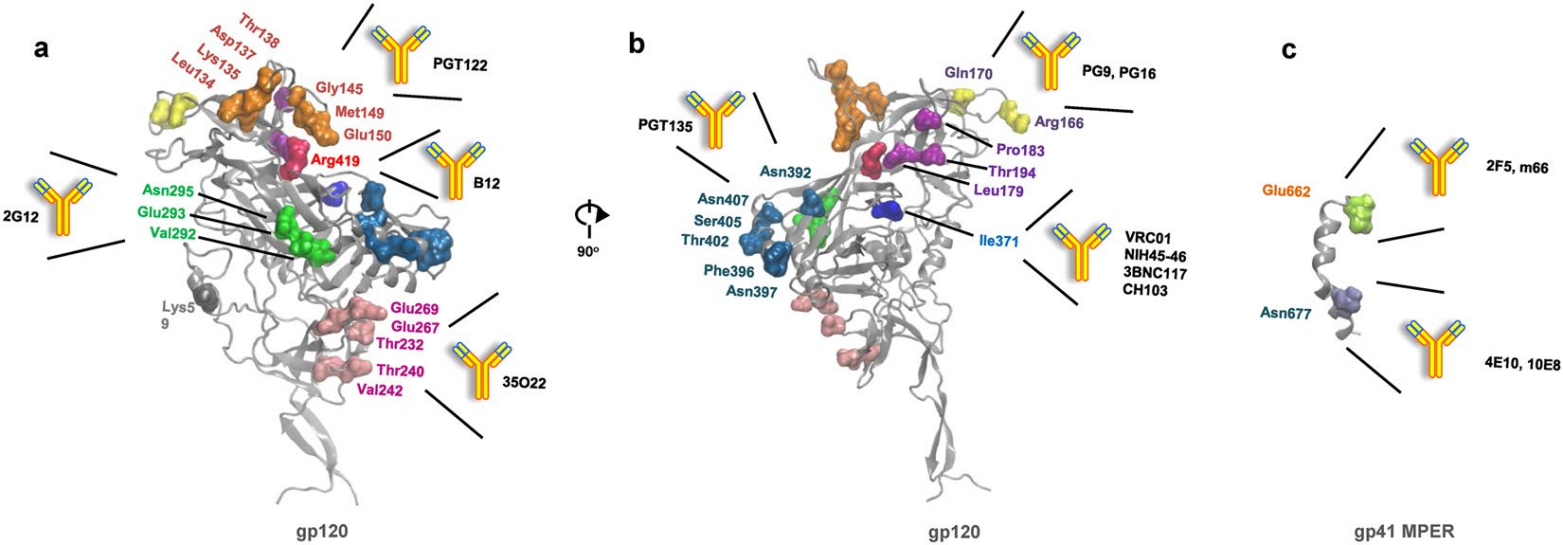
Mapping of treatment failure-related mutations on the high-resolution structures of HIV-1 gp120 (**a**) and 90° views (**b**) and gp41 MPER (**c**). Most treatment failure-related mutations overlapped with the neutralization epitopes of different kinds of bnAbs.

To better understand the mutations identified by bioinformatic prediction, we mapped these residues on the high-resolution structures of HIV-1 gp120 (PDB ID 4TVP) and gp41 (PDB ID 2PV6). To our surprise, almost all of these residues (28 of 31 in gp120 exterior Env and 2 of 3 in the outer membrane part of gp41) appear to be involved with possible epitopes of previously reported neutralizing antibodies (Fig. 3). In the gp120 region, residues 134, 135, 137, 138, 145, 149 and 150 are located in a small flexible loop of the V1/V2 region which is accessible to a number of V1/V2-directed antibodies^20–23^. For example, the bnAb PGT122 recognizes glycan and protein elements in both V3 and V1/V2 region, including residues in the 134–150 loop^22^. Residues 166 and 170 are located in another area of the V1/V2 region and can be targeted by another panel of neutralizing antibodies. The bnAbs PG9 and PG16 have direct interaction with the gp120 residue 170^24, 25^. The surface area where residues 179, 183 and 194 are located is part of the epitopes of mAb 830A^26^. Residues 232, 240, 242, 267 and 269 form a surface that can be targeted by the 35O22-like bnAbs, and 35O22 has direct interaction with gp120 residue 240^27^. The surface comprised of residues 292, 293 and 295 can also be accessed by neutralizing antibodies, such as mAb b12^28^. Residue 371 is located in the CD4-binding site and has direct interaction with a number of bnAbs, including VRC-class antibodies (e.g., VRC01, VRC06, VRC07 and VRC23)^29–31^, CH103^32^, b12 and F105^33^, NIH45-46^34^, 12a21^35^, and 3bnc117^36^. The surface area where residues 392, 396, 397, 402, 405 and 407 are located can be accessed by some glycan-dependent bnAbs. For example, bnAb PGT135 recognizes the glycosylation site Asn392^37^. Residue 419 also interacts with some neutralizing antibodies, including b12, 17b^37^ and X5^38^.

In gp41, 2 of the 3 identified residues are located in the highly conserved membrane-proximal external (MPER) region in the outer membrane part of gp41. Residue 662 can directly interact with bnAbs 2F5 and m66^39, 40^, while residue 677 can interact with bnAbs 4E10 and 10E8^41, 42^. Taken together, these results indicate a significant correlation between treatment failure-related mutations and the epitopes of HIV-1 neutralizing antibodies.

### Molecular docking

**Figure 4:**
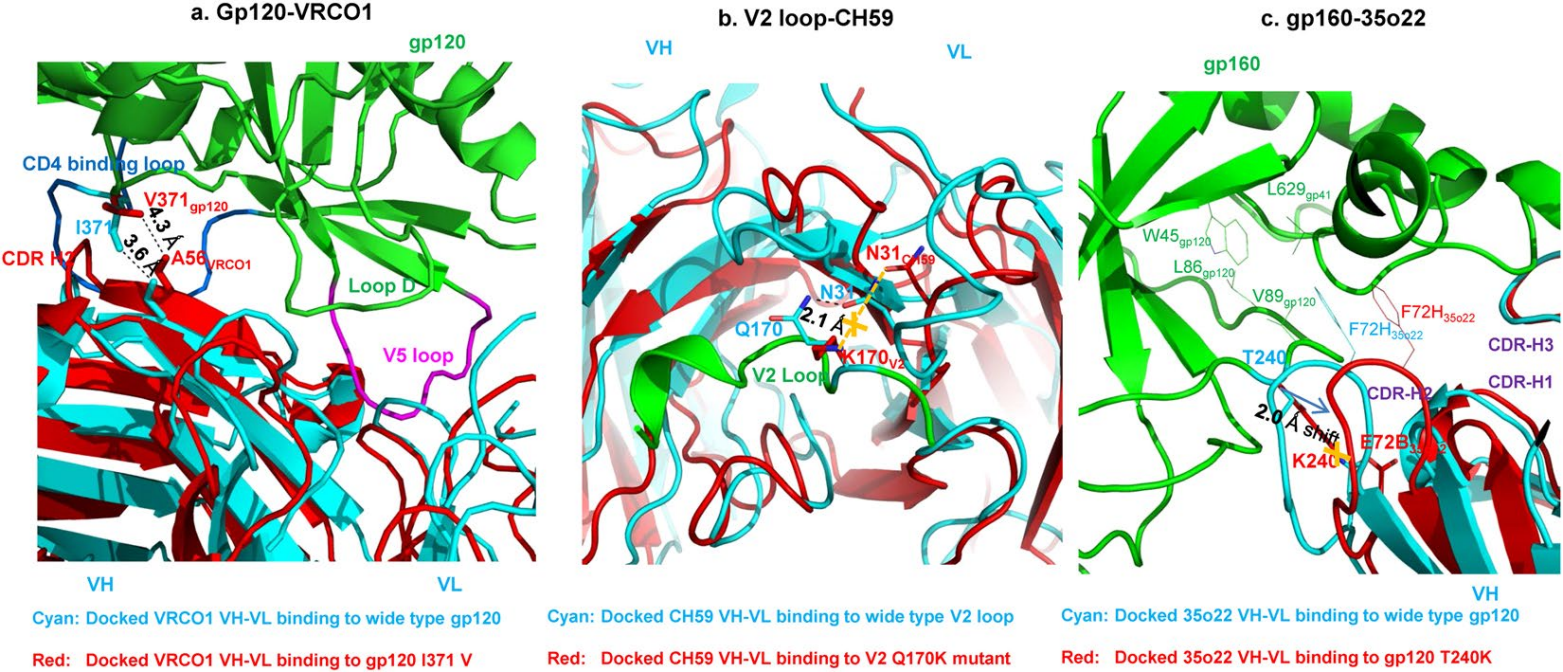
Molecular docking results showed the impact of adaptive mutations of HIV-1 Env on the binding ability of bnAbs. (**a**) VRC01 was colored in cyan in the docked VRC01 VH-VL binding to wild-type gp120 and colored in red in the docked VRC01 VH-VL binding to gp120 I371 V. (**b**) CH59 was colored in cyan in the docked CH59 VH-VL binding to wild-type gp120 V2 loop and colored in red in the docked CH59 VH-VL binding to gp120 V2 Q170K mutant. (**c**) 35O22 was colored in cyan in the docked 35O22 VH-VL binding to wild-type gp120 and colored in red in the docked 35O22 VH-VL binding to gp120 T240K mutant.

We next employed molecular docking to investigate if the treatment failure-related adaptive mutations of HIV-1 Env would compromise the binding capability of bnAbs. By using the Z-DOCK program^43^, three relatively high-frequency mutations, including I371V in gp120 CD4-binding loop within the epitope of VRC01; Q170K in gp120 V2 loop within the epitope of CH59; and T240K in gp120 seven-stranded sheets within the epitope of 35O22, were evaluated to determine how extensively these mutations could affect antibody-gp120 binding. For the docked VRC01/gp120 binding^36^, a Z-rank was obtained with a top pose score of −120.767 for VRC01-binding to wild-type gp120, in contrast to the score of −112.569 for binding to mutant gp120 I371V, indicating that the binding of VRC01/gp120 encounters a more energetically favorable docking compared to the binding of VRC01/gp120-I371V. In detail, VRC01 VH moved closer to the gp120 CD4-binding loop by ~1 Å shift in response to the I371 to V371 mutation (Fig. 4a), resulting in a conformation of VRC01 VH more like that of CD4, and the VRC01 VL disengaged from V5 loop to release steric clash with gp120 inner domain core. Consequently, the clasped V5 loop at the VRC01 VH/VL interface, which constitutes an important factor for V5 sequence variation tolerance of VRC01 neutralization breadth^29^, became more solvent-exposed in the gp120 I371V mutant. Thus, it is possible that the breadth and potency of VRC01 may be compromised in neutralizing HIV-1 bearing the gp120 I371V mutation. In addition, we found that I371_gp120_ is close to A56_VRC01_ with a distance of 3.6 Å for VRC01/gp120, indicating an σ-σ super-conjugation interaction which is compromised in VRC01/gp120-I371V with a longer distance of 4.3 Å. Similarly, for the docked CH59/gp120 complex^44^, CH59 binds more favorably to wild-type gp120 than to gp120 with Q170K mutation (Z-score of −109.932 for wild type *vs*. −86.583 for Q170K mutant). Notably, the strong H-bond of Q170_gp120_ NE2-N31_CH59_ OD1, which exists at the CH59/gp120 interface with a distance of 2.1 Å and is the major non-bonding interaction for associating CH59 and gp120 V2 peptide, was found to be broken in the CH59/gp120-Q170K complex (Fig. 4b). For the docked 35O22/gp160 binding^27^, the H-bond of T240_gp120_-E72_35O22_ in 35O22/gp160 complex was evidently perturbed by T240 to K240 mutation, in which the long side chain of lysine elicits substantial steric clash with the glutamine carbonate group of E72_35O22_. Consequently, to facilitate 35O22 binding, the gp120 T240K mutant needs to expel the protruding VH CDR2 loop of 35O22 by a ~2.1 Å shift from the binding cleft formed by the gp120 seven-stranded sheets group and gp41 α8 helix (Fig. 4c). Such change would compromise the hydrophobic interactions, consisting of F72H_35O22_ and W45_gp120_, L86_gp120_, and V89_gp120_, as observed in the 35O22/gp160 complex. Thus, the binding of 35O22/gp160 encounters more energetically favorable docking than the binding of 35O22/gp160-T240K (Z-score of −52.893 *vs*. −36.811). Based on the docked structural analysis of these three treatment failure-related mutations in Env proteins, antibody binding ability was compromised, irrespective of epitope location.

### Virus sensitivity to the bnAbs

The neutralization assay was performed to determine whether the adaptive mutation would affect the sensitivity of mutant-viruses to the bnAbs. The IC_50_ of the bnAb VRC01 against the mutant viruses (I371V) was assessed and compared with that against the wide-type viruses (AT1). Indeed, we found that the I371V mutant virus gained 4.9 times enhancement of resistance to VRC01 (IC_50_ = 0.0724 ± 0.0012 μg/ml for I371V, and IC_50_ = 0.0148 ± 0.0009 μg/ml for AT1). This result suggests that some mutant viruses in the treatment-failure patients may have acquired resistance to some bnAbs.

## Discussion

A thorough understanding of humoral immune responses to HIV-1 has been a major research focus in recent years^45^. HIV-1 has been reported to escape neutralizing antibodies during early infection via a “glycan shield” mechanism whereby escape mutations in the Env become involved in N-linked glycosylation; such mutations were very sparse and, generally, did not map to known neutralization epitopes^11^. Humoral responses also evolve in response to these Env escape mutations, but the kinetics of evolution does not correlate with the rate of disease progression^6^. Recently identified broadly neutralizing antibodies (bnAbs) against some conserved epitopes have rekindled interest in exploring antibody-virus co-evolution pathways *in vivo*, but these antibodies only naturally arise in a fraction of individuals after years of HIV-1 infection. Thus, despite intensive research, important gaps remain in our understanding of humoral immune responses relative to HIV-1 disease progression. In particular, considering that selection pressure exerted on viruses has been largely replaced by antiretroviral drugs in ART-treated patients, it is important to ascertain whether and how humoral immune responses affect disease progression under this circumstance.

In the present study, gene sequencing and selection pressure-based analysis were employed to uncover the underlying relationships between treatment failure and putative adaptive mutations occurring in the HIV-1 *env* gene. A study population was very carefully selected for homogeneity in terms of demographics, ethnic makeup, treatment modality and, importantly, route of infection (infected with the same HIV-1 subtype B’ via former plasma donation). This unique feature enabled a comparative study across subpopulations that exhibited different treatment outcomes. Interestingly, although different baseline variables of the TF and VS groups were compared, including, for example, CD4^+^ T cell count, viral load, drug-resistant mutations, and co-receptor usage, the only variable that showed a significant difference between the two groups was gp160 diversity. The gp120/gp41 diversity did not increase or decrease in these TF patients during long-term ART, suggesting that the viruses may have evolved to escape host immune responses before the initiation of ART via quasispecies diversification. This also suggests that the level of gp160 diversity may be used as an indicator to predict disease prognosis under certain circumstances.

The residues that underwent positive selection pressures were determined by calculation of Ka/Ks ratio for individual amino acids in gp120 or gp41. Based on the crystal structures of gp120 and gp41 proteins, we mapped these residues onto the structures of Env. In contrast to the untreated patients in which Env escape mutations were sparse and could not be mapped to known neutralization epitopes, we found that most of these mutation sites overlapped with the neutralization epitopes of different kinds of bnAbs: R419K and I371V locating in the epitope of CD4bs antibody b12 and VRC01; R166Q and Q170K in the epitope of V1/V2 targeting antibodies PG9, PG16, and CH59; T232R, T240K, V242I, E267K, E269G in the epitope of gp120/gp41 interface antibody 35O22; E662K and N677K in the epitope of MPER antibody 2F5 and 4E10 (Fig. 3). These results demonstrate, for the first time, that, to the best of our knowledge, HIV-1 escape from host humoral immunity contributes to disease progression in patients on long-term ART.

Our analysis may have important implications concerning the proposition of an Ab effect on the virological outcome after HAART treatment. For instance, we did not find differences in the number of PNG and length of V3 loop sequence between TF and VS patients, suggesting that the glycosylation sites were not triggered by the immune response in these patients, and also that the V3 loop epitope may not be relevant for immune escape. The highest level of mutation was located in the C2 region of Env, suggesting that this region may be one of the most interesting epitopes to target. Notably, our analysis was based on the published crystal structures of bnAbs complexed with gp120. Due to the limited complex structures available, it is probably that some of the mutations are located within epitopes that have not been structurally characterized, or the Abs targeting them have not been identified. For example, the 134–138 loop (Leu134, Lys135, Asp137, Thr138) are just located on the Env surfaces and could be a dominant epitope by a number of Abs, but we only found one bnAb, PGT122, that mostly recognizes V3 but also has some interactions with the 134–138 loop. We believe that further studies, e.g., by using B cells from the patients described in this study, could lead to the isolation of Abs targeting this loop.

To elucidate the mechanism that governs the development of these treatment failure-related adaptive mutations, we selected three bnAbs (VRC01, 35O22, and CH59) that have available crystal structures in complex with gp120/gp160 for computational modeling analysis to evaluate whether Env adaptive mutations have an impact on antibody binding. The docking results suggest that these mutations tend to compromise antibody-antigen interactions. While the accumulation of HIV-1 escape mutations is a very complex process, this finding suggests that escape of antibody binding could be one of the routes via which the viruses escape from host humoral immunity in long-term antiretroviral-experienced patients. In a proof-of-concept study, we generated a mutant HIV-1 virus with a single I371V mutation, and compared side-by-side the neutralization activity of VRC01, the most advanced clinically developed HIV-1 bnAbs, against the wild-type and the mutant virus. The HIV-1 I371V mutant gained 4.9 times enhancement of resistance to VRC01, suggesting that some viruses from TF patients may have acquired resistance to some bnAbs. Nevertheless, further study is warranted to investigate how the emergences of these HIV-1 mutants cause the ART failure. For instance, we will isolate these viral mutants and test their sensitivity to the antiretroviral drugs used by the TF patients, even though these drugs are not expected to target the HIV-1 Env, because we cannot exclude the possibility that the mutations of viral Env may cause the change of viral replication kinetics and viral sensitivity to some of the antiretroviral drugs.

These findings have considerable importance for developing an effective long-term antiviral strategy against HIV-1. Although highly-active ART (HAART) is very effective in suppressing viral replication, it can result in toxicity with unpredictable effects and resistance to therapy, especially with long-term use, resulting in treatment failure in a substantial proportion of individuals^2, 4, 5^. Of note, advances in antibody-based therapeutics are coming at an ever-increasing pace, and, as a result HIV-1 bnAbs, hold promise in the ongoing effort to eradicate HIV^20, 46–49^. In the present study, it was shown that host humoral immunity may play a role in treatment failure in long-term ART-treated patients. Some mutations in HIV-1 gp120/41 may have resulted in escape from some neutralizing antibodies and thus contributed to disease progression. This implies that a combination of conventional antiretroviral drugs with potent bnAbs, especially a cocktail of bnAbs targeting different epitopes, could be beneficial in patients who have shown poor response to ART. Moreover, given that there is no mutation in the HIV-1 gp41 NHR region, the target of HIV-1 fusion inhibitors, the combination of T20 with antiretroviral drugs may be provided to the patients immediately after their viruses are identified to carry the mutations in the sites overlapped with the neutralization epitopes of bnAbs, to avoid the treatment failure.

In conclusion, our study provides evidence that escape from host humoral immunity may play a role in treatment failure in long-term antiretroviral-experienced patients. Treatment failure-related *env* mutations identified by SGA sequencing and selection pressure-based analysis may provide an immunological basis for understanding the co-evolution of viruses and bnAbs in a particular patient. Based on *env* gene information of the viruses in the patients, we may be able to predict the treatment outcome, based on which individualized therapeutic regimen, such as the combinations of antiretroviral drugs with HIV-1 entry inhibitors targeting the coreceptor (e.g., maraviroc) or gp41 (e.g., enfuvirtide), rather than gp120 where the mutations are located, may be designed in advance for the patients with TF potential.

## Methods

### Ethics statement

This study was approved by the Institutional Research Ethics Community of the Chinese Center for Disease Control and Prevention, and all subjects signed informed consent to participate in the research study prior to blood and data collection. All experiments were performed in accordance with relevant guidelines and regulations.

### Study patients

The study population consisted of 45 chronically HIV-infected patients on a stable ART regimen who participated in a multicenter AIDS Cohort Study in Anhui and Henan provinces in China from 2004 to 2011. Patients who initially received therapy with a first-line regimen of lamivudine (3TC), stavudine (d4T), or zidovudine (AZT) and nevirapine (NVP) or efavirenz (EFV) were selected. Patients in this study met the following inclusion criteria: be naïve-ART before enrollment and be 18–65 years old with uninterrupted HAART for more than 12 months. 3 patients in TF group and 2 patients in VS group who were lost to follow-up at the last sampling point were excluded. Blood samples collected at each follow-up point were analyzed for viral load, CD4^+^ T cell count and drug-resistance mutations. Plasma viral loads were determined with real-time Nucleic Acid Sequence Based Amplification (NASBA) (NucliSense Easy Q, BioMerieux, France) according to the manufacturer’s instructions. CD4^+^ T cells in whole blood were quantitated by flow cytometry using Tritest CD3/CD4/CD45 TruCount Tubes (BD Biosciences, San Jose, CA). For detection of drug-resistance mutations, viral RNA was first extracted from plasma using the QIAamp® Viral RNA Mini Kit (Qiagen, Chatsworth, CA) according to the manufacturer’s protocol and then used to synthesize first-strand cDNA using the SuperScript™ III First-Strand Synthesis System (Invitrogen). The HIV-1 pol gene was amplified with specific primers that annealed to conserved regions of the pol region, and sequences of the pol gene were compared to the consensus B reference sequence using HIVdb software (Stanford HIV Drug Resistance Database, http://hivdb.stanford.edu) to detect the drug-resistance mutations.

### cDNA synthesis, single-genome amplification and HIV-1 *env* sequencing

HIV-1 RNA extraction, cDNA synthesis, and SGA were performed as previously reported^50^. For SGA, according to a Poisson distribution, a positive reaction rate of 30% or lower, or cDNA diluted to ensure fewer than 29 PCRs yielded from a total of 96 independent PCRs thus ensuring that amplicons were derived from a single template. Sequences of the *env* region were amplified by a nested polymerase chain reaction using Premix Ex Taq version 2.0 (Takara, Japan). PCR products derived from cDNA dilutions yielding less than 30% PCR positivity were sequenced by an ABI 3730XL Sequencer using BigDye terminators (Applied Biosystems, Foster City, California, USA). The chromatogram data were cleaned and assembled using Sequencher v4.9 (Gene Codes, Ann Arbor, Michigan, USA).

### Sequence analysis

Multiple nucleotide sequence alignments of gene sequences encoding for the Env CDS, V1V2, V3, V4 and V5 of HIV-1 from each subject were conducted using Clustal W, input in Gene Cutter tool from the Los Alamos HIV sequence database (http://www.hiv.lanl.gov/content/sequence/GENE_CUTTER/cutter.html) and then adjusted manually with BioEdit software (version 7.1.3). Phylogenetic analysis was performed using the Kimura-2 model of substitution within neighbor-joining method in MEGA 5 at the nucleotide level, and the reliability of the branching orders was tested by bootstrap analysis of 1,000 replicates^51^. HIV-1 co-receptor usage (CCR5 and CXCR4) was predicted using WEBPSSM (http://indra.mullins.microbiol.washington.edu/webpssm/). Sequence diversity was calculated using the Kimura 2-parameter model option in MEGA 5. The number and location of potential N-linked glycosylation sites (PNGSs) for each sequence were estimated using the N-GLYCOSITE web tool from the Los Alamos HIV database (http://www.hiv.lanl.gov/content/sequence/GLYCOSITE/glycosite.html).

### Identification of putative adaptive mutations directly related to treatment failure

A total of 638 *env* sequences, including 352 sequences in the VS group and 286 sequences in TF group, acquired from patients samples before treatment (baseline) were analyzed to screen treatment failure in relation to adaptive mutation by a selection pressure-based analysis. The Ka/Ks ratio could be used as an indicator of selective pressure acting on a protein-coding gene. The computation of Ka/Ks for an individual site or specific amino acid was based on the model of Chen *et al*.^52^. A Ka/Ks value of 1, >1 or <1 indicated a neutral selection, a positive selection or a negative selection, respectively. Log odds (LOD) confidence score was used to measure the significance of selection pressure, and LOD > 2 indicated a significance of the positive selection. Ka/Ks and LOD were calculated by the CorMut R package^53^. When performing the computation, HXB2 served as a reference.

Bayesian network was applied to further analyze the correlation between predicted mutations and treatment outcome. Generally, the Bayesian network is a graphical model that encodes a joint probability distribution of a set of random variables, where nodes represent random variables, and arrows represent probabilistic dependencies between them^54^. The Bayesian network was constructed by the bnlearn R package, and the results were revised by the nonparametric bootstrap method. More than 65% of bootstrap support was considered robust, while 35–65% was considered thin^55^. The mutations having direct connection with treatment failure with bootstrap value over 65% were identified.

### Virus growth competition assay

A multiple-cycle recombinant virus growth competition assay was applied to determine the impact of quasispecies variation on viral fitness. Mutations were introduced to the wild-type plasmid pAT1 by PCR to construct the mutant plasmids which contained the critical adaptive mutation sites. Then HEK-293T cells were transfected with mutant plasmids to rescue the viruses with adaptive mutations. TCID_50_ of viruses with adaptive mutations was quantitated to detect viral infection ability. Mutant virus and wild-type virus AT2 co-infected PM1 cells with equal P24 antigen levels. Cells were collected from 3 to 6 days post-infection. Cells were stained by anti-Thy1.1 antibody and anti-Thy1.2 antibody, respectively, and subsequently both antibodies. The ratio of the two viruses was detected by FACS. Relative fitness was calculated using the online tool VFitness (http://bis.urmc.rochester.edu/VFitness/FitnessTwo.aspx).

### Molecular docking

The Z-DOCK program^43^ implanted in the Accelrys Discovery Studio (http://accelrys.com) was used to fit bnAbs VRC01, CH59 and 35O22 onto gp120. The output Z-scores for the top poses was used to determine the effects of treatment failure-related residues of Env proteins on antibody binding. The three-dimensional structures for these Fab and HIV Env were extracted from the resolved crystal structure of VRC01/gp120 (PDB ID 4LST)^36^, CH59/gp120 (PDB ID 4HPY)^44^, and 35O22/gp160 complexes (PDB ID 4TVP)^27^. For calculation convenience, the constant regions of Fab were removed with only consideration of the V regions (VH-VL). Env mutants, including I371V for VRC01, Q170K for CH59, T240K for 35O22, were modeled by the PyMOL molecular graphics system (version 1.5.0.4; Schrödinger, LLC) without atomic clashes.

Before docking, the structures of bnAbs and Env were processed as follows. 1) water molecules were deleted and hydrogen atoms were added at pH7.4 and an ionic strength of 0.145 and in a dielectric environment of 10; 2) energy was minimized based on CHARMM^56^ with a cutoff of 0.9; 3) loop regions were rebuilt according to SEQRES^57^ data; and 4) cysteine bridges in these proteins were defined as blocked regions. Then Z-DOCK performed a systematic search of a uniform sample of docked protein poses and used an internal scoring algorithm to predict optimal interactions. A total of 54000 docked poses were initially produced, which were further filtered and re-ranked to obtain the top 200 poses based on Z-Rank score using electrostatic and desolvation energy and non-deterministic FFT optimization. These 200 poses were then visually scrutinized and judged by Z-Score. The most reasonable poses were chosen as the models for the VH-VL-Env complexes by combined consideration of general features of antibody-antigen interaction, such as hydrogen bonds, salt bridges, hydrophobic packing and other interactions at the interface, without any highly unusual features and steric clashes. The Z-scores for VH-VL docking into wild-type and mutant Envs were evaluated and compared.

### Neutralization assay in TZM-bl cells

A sensitive HIV-1 neutralization assay was performed to test the neutralizing activities of the bnAb VRC01 against HIV-1 carrying Env with I371V mutation as previously described^58^. Briefly, the bnAb was at serial dilution was incubated with the mutant virus (200 TCID_50_) for 1 h at 37 °C. The virus with wild Env sequence (AT1) was included as control. TZM-bl cells (1 × 10^4^) with DEAE-dextran (15 μg/ml) were added to each well. After 48 h incubation, The RLUs were determined using the luciferase reporter gene assay system (Bright-Glo; Promega) and the luminometer (PerkinElmer Life Sciences), as described previously^58^. Mock infected cells were used to determine background luminescence. IC_50_ (the concentration of an inhibitor causing 50% inhibition of infection) value was then calculated^59^.

### Statistical analyses

Comparisons of differences between VS and TF groups were calculated using Mann-Whitney, Chi-Square or Fisher’s exact test. The relative fitness comparison between mutant viruses and wild-type viruses was done using the Wilcoxon rank sum test. Statistical significance was set at P value < 0.05 and for two-sided tests. Logistic regression and receiver operating characteristic (ROC) analysis were performed to develop a predictive model. Covariates, including CD4^+^ T cell count, viral load, gp160 diversity, drug-resistant mutations and co-receptor usage, were included for logistic analysis. The statistical analyses were performed using the SPSS software package (version 17.0).

## Electronic supplementary material

Supplementary Tables
