# Supplementary material for: Escape from humoral immunity is associated with treatment failure in HIV-1-infected patients receiving long-term antiretroviral therapy
Source: Sci Rep. 2017 Jul 24;7:6222. doi: 10.1038/s41598-017-05594-5 (PMC5524822; doi:10.1038/s41598-017-05594-5)
Supplement: Supplementary file 1 — Supplementary Tables [file 41598_2017_5594_MOESM1_ESM.doc]

**Title:** Escape from humoral immunity is associated with treatment failure in HIV-1-infected patients receiving long-term antiretroviral therapy

**Authors’ names:** Yabo Ouyang, ^1,5#^ Qianqian Yin, ^1#^ Wei Li, ^2,3^ Zhenpeng Li, ^1^ Desheng Kong, ^1^ Yanling Wu, ^2^ Kunxue Hong, ^1^ Hui Xing, ^1^ Yiming Shao, ^1^ Shibo Jiang, ^2,4*^ Tianlei Ying, ^2*^ Liying Ma^1*^

**Table S1. Sequence characteristics of amino acid length and potential N-linked glycosylation sites**

|  | AA length  Mean (Median, Range) | | |  | No. of PNGs  Mean (Median, Range) | | |
| --- | --- | --- | --- | --- | --- | --- | --- |
|  | VS | TF | *P*^a^ |  | VS | TF | *P*^a^ |
| gp160 | 856.72 (856, 837-887) | 856.55 (855, 839-882) | 0783 |  | 32.11 (33, 26-39) | 32.16 (32, 24-39) | 0.799 |
| gp120 | 509.89 (509, 489-541) | 510.82 (508, 492-535) | 0.191 |  | 27.31 (28, 21-34) | 27.31 (27, 19-34) | 0.891 |
| gp41 | 346.83^$^  (347, 343-350) | 345.85 (347, 337-347) | 0.000^**^ |  | 4.81  (5, 4-6) | 4.85  (5, 3-6) | 0.138 |
| C1 | 128.95 (129, 123-130) | 129.09 (129, 117-133) | 0.488 |  | 1.95^$^  (2, 1-3) | 1.87  (2, 1-4) | 0.041^*^ |
| V1V2 | 70.07 (68, 57-97) | 70.10 (67, 59-93) | 0.603 |  | 6.84 (6, 3-12) | 7.03  (7, 4-11) | 0.331 |
| C2 | 99.00 (99, 99-99) | 99.00 (99, 99-99) | 1.000 |  | 7.03^$^  (7, 5-8) | 6.82  (7, 4-8) | 0.039^*^ |
| V3 | 34.95 (35, 27-35) | 34.96 (35, 29-35) | 0.828 |  | 0.97  (1, 0-1) | 1.02^$^  (1, 0-2) | 0.000^**^ |
| C3 | 52.03 (52, 46-54) | 52.20^$^  (52, 44-54) | 0.000^**^ |  | 3.19  (3, 2-4) | 3.45^$^  (4, 0-4) | 0.000^**^ |
| V4 | 30.55 (30, 22-42) | 31.15 (30, 22-41) | 0.072 |  | 4.56  (5, 2-6) | 4.57  (5, 2-6) | 0.502 |
| C4 | 42.47^$^  (42, 41-50) | 42.44 (42, 30-51) | 0.031^*^ |  | 1.06^$^  (1, 0-2) | 0.90  (1, 0-2) | 0.000^**^ |
| V5 | 11.98^$^  (12, 9-12) | 11.93  (12, 9-12) | 0.035^*^ |  | 1.70  (2, 1-3) | 1.65  (2, 0-3) | 0.197 |
| C5 | 39.99 (40, 38-40) | 39.98 (40, 34-40) | 0.428 |  | 0.00  (0, 0-0) | 0.00  (0, 0-0) | 1.000 |

^a^ Mann-Whitney Test; ^$^ Mean Rank group with higher value; ^*^*P* < 0.05; ^**^*P* < 0.01

**Table S2. Potential adaptive mutations directly related to treatment failure**

| Mutation Site^a^ | Region | Population frequency^b^ | | Sequence frequency | | TF. Population  frequency | | |
| --- | --- | --- | --- | --- | --- | --- | --- | --- |
|  |  | TF | VS | TF | VS | T_0_ | T_1_ | T_2_ |
| G145N | V1/V2 | 0.5 | 0.08 | 0.2 | 0.01 | 0.583 | 0.417 | 0.5 |
| E150S | V1/V2 | 0.15 | 0 | 0.14 | - | 0.167 | 0.083 | 0.083 |
| I371V | C3 | 0.55 | 0.04 | 0.21 | 0 | 0.75 | 0.583 | 0.417 |
| N392S | V4 | 0.2 | 0.04 | 0.14 | 0 | 0.083 | 0.083 | 0.167 |
| A607N | gp41 | 0.15 | 0 | 0.16 | - | 0.083 | 0.083 | 0.083 |
| E662K | gp41 | 0.35 | 0 | 0.21 | - | 0.333 | 0.333 | 0.333 |
| N677K | gp41 | 0.8 | 0.44 | 0.61 | 0.27 | 0.917 | 0.75 | 0.75 |
| E734G | gp41 | 0.2 | 0.04 | 0.12 | 0 | 0.083 | - | 0.167 |
| V832L | gp41 | 0.15 | 0 | 0.08 | - | 0.167 | 0.167 | 0.25 |

^a^ HXB2 gp160 was used as a reference sequence. ^b^ Population frequency at baseline.

**Table S3. Number of sequences of 12 patients included in the longitudinal Analysis**

| Sample | Sequences no. | | |
| --- | --- | --- | --- |
|  | T0 | T1 | T2 |
| 2039 | 21 | 12 | 10 |
| 2124 | 11 | 5 | 14 |
| 2142 | 10 | 12 | 26 |
| 2243 | 2 | 9 | 22 |
| 2358 | 16 | 19 | 11 |
| 2360 | 5 | 18 | 11 |
| 2361 | 19 | 8 | 18 |
| 2362 | 25 | 10 | 23 |
| 2408 | 18 | 15 | 13 |
| 2418 | 16 | 30 | 20 |
| 3124 | 7 | 17 | 14 |
| 3954 | 19 | 16 | 14 |
